# Prostate Cancer: From Genomics to the Whole Body and Beyond

**DOI:** 10.1155/2017/8707690

**Published:** 2017-11-09

**Authors:** Fabio Grizzi, Gianluigi Taverna, Richard J. Cote, Giorgio Guazzoni

**Affiliations:** ^1^Department of Immunology and Inflammation, Humanitas Clinical and Research Center, Rozzano, 20089 Milan, Italy; ^2^Department of Urology, Humanitas Clinical and Research Center, Rozzano, 20089 Milan, Italy; ^3^Department of Urology, Humanitas Mater Domini, Castellanza, 21053 Varese, Italy; ^4^Department of Pathology and Laboratory Medicine, University of Miami Miller School of Medicine, Miami, FL 33136, USA; ^5^Sylvester Comprehensive Cancer Center, University of Miami Miller School of Medicine, Miami, FL 33136, USA; ^6^Department of Biochemistry, University of Miami Miller School of Medicine, Miami, FL 33136, USA

Prostate cancer represents the second most common cancer in men globally [1]. Prostate, lung and bronchus, and colorectal cancers account for 44% of all cases in men, with prostate cancer alone accounting for 1 in 5 new diagnoses. Despite rapid advances in the fields of molecular and cell biology, how neoplastic prostate cells progress through carcinogenesis remains widely debated.

Prostate cancer is recognized as a complex and multifactorial dynamical disease that is discontinuous in* space* and* time*, but advances through qualitatively different states. It is known that prostate cancer is characterized by a high degree of pathological and genetic heterogeneity compared to other human cancers. Recently, several studies have investigated the molecular basis of primary prostate cancer and have identified recurrent genomic alterations, including mutations, DNA copy-number changes, gene rearrangements, and gene fusions [2–4]. Heterogeneous genomic aberrations may lead to prostate cancer onset, disease progression, and metastatic potential. This heterogeneity may also contribute to the variable drug responses observed among affected patients. Serum Prostate-Specific Antigen (PSA) is, currently, the most important biomarker for the detection, follow-up, and therapeutic monitoring of prostate cancer. PSA based screening for prostate cancer has had an important impact on the epidemiology of the disease. Its use has been associated with a significant reduction in prostate cancer mortality, but has also resulted in the overdiagnosis and overtreatment of indolent prostate cancer, exposing many men to treatments without benefits [5]. Its low specificity and sensitivity are mainly attributable to the fact that serum PSA may also be increased in benign conditions, such as benign prostatic hyperplasia and chronic prostatitis. Additionally, serum PSA levels are affected by biologic variability that may be related to differences in androgen levels or prostate manipulation and may have distinct racial variation [6]. Ludwig et al. recently reported that men with an undetectable serum PSA 20 years after radical prostatectomy had a very low rate of recurrence and no deaths due to prostate cancer, suggesting that 20 years is a reasonable time to discontinue PSA testing [7]. Given that an elevated PSA can be difficult to interpret, in the last decade proteomic and genomic technologies have been applied as powerful ways to uncover biomarkers of detection, prognosis, and prediction and to improve the understanding of prostate cancer biology. Several investigators have proposed alternative biomarkers that include the [−2]proPSA isoform [also called prostate health index (PHI)], the 4K score, a combination of four kallikrein proteins, and immunological or genomic biomarkers. It has been proposed that biomarkers for prostate cancer may be roughly classified in five categories based on their origin: genome, epigenome, transcriptome, proteome, or metabolome. Circulating tumor cells (CTCs) have been detected in different epithelial cancer types and have emerged as promising prognostic biomarkers [8]. Furthermore, the discovery of microRNAs (miRNAs) has led to investigating this class of small noncoding RNAs as new biomarkers for prostate cancer detection and prognosis. However, due to the small quantity of these molecules and the lack of standard strategies for normalization and validation as well as the high degree of inconsistency among studies, the discovery of such biomarkers is still challenging. The study of prostate cancer metabolism represents another topic of great interest to understand the mechanisms underlying the development and progression of prostate cancer [9]. These metabolic features are of clinical interest as they present a variety of potential therapeutic targets.

Alternative screening strategies have also been proposed. Actually, nearly 90% of prostate cancers are clinically localized at the time of their diagnosis. The clinical behavior of localized prostate cancer is, however, highly variable. Some men will have aggressive cancer leading to metastasis and death from the disease while others will have indolent cancers that are cured with initial therapy or may be safely observed. Multiple risk stratification systems have been developed, combining the best currently available clinical and pathological parameters that include the digital rectal examinations, serum PSA levels histological Gleason score, and clinical and pathological staging; however, these tools still do not adequately predict outcome. Today, the diagnosis of prostate cancer remains based primarily on the microscopic observation of prostate tissue sampled throughout needle biopsy. Conventionally, a systematic prostate biopsy is performed using transrectal ultrasound to obtain 10 to 12 tissue cores. Even though systematic prostate biopsy represents the standard strategy, this approach misses 21% to 28% of prostate cancers and undergrads 14% to 17% [10–12]. Although pathologic grading and staging is one of the strongest predictors of prostate cancer outcome, recent changes to Gleason score assignment have improved the risk stratification and reproducibility of grading. There is great potential, however, for further improvement/optimization based on specific histological features that are not currently accounted for by the Gleason scoring systems and by additional quantitative analysis. Even more sophisticated and precise imaging tools also have been introduced to enhance diagnostic performance. Multiparametric magnetic resonance imaging/ultrasound fusion biopsy has been reported as a tool able to improve detection of high-grade cancers when compared to systematic biopsy. Furthermore, it has been shown that Positron Emission Tomography/Computed Tomography (PET/CT) and whole body magnetic resonance imaging (MRI) scans have the potential to improve detection and to assess response to treatment of all states of advanced prostate cancer. However, standardization of acquisition, interpretation, and reporting of whole body (WB) MRI and PET/CT scans is required to assess the performance of these techniques in clinical trials of treatment approaches in advanced prostate cancer.

Cancer research has generated an intricate “body of knowledge” showing that prostate cancer is a disease that involves dynamic changes in the genome. Further risk stratification using molecular features could potentially help distinguish indolent from aggressive prostate cancer. Further studies are also needed to investigate the potential predictive value of this procedure to identify prostate cancer. Additionally, circulating tumor cells and cell-free circulating tumor DNA in the blood have emerged as potential promising tumor avatars. microRNAs and the study of the prostate cancer metabolism are further attractive areas of research. Recently, it has been demonstrated that a trained canine olfactory system can detect prostate cancer specific volatile organic compounds (VOCs) in urine samples with high estimated sensitivity and specificity [13]. This approach might have the potential to offer a noninvasive alternative to PSA sampling and prostate biopsy for detecting prostate cancer. In addition, the results suggest that prostate cancer specific VOCs might depend on a metabolic process of the tumor.

It is now ascertained that prostate cancer is governed by a “multiscale causality.” This not only recognizes multiple processes and controls acting at multiple scales, that is, from the gene level [2] to that of organism with neurobiological and psychological evidences [14, 15], but, unlike a strict reductionist approach, may also recognize the fact that relevant “first principles” may reside at scales other than the smallest microscales. In other words, the observed phenomenon at each level of biological organization, that is, scale, has structural and behavioral proprieties that do not exist at lower or higher organizational levels. In addition, although each of the spatial scales may have multiple temporal scales, biological process that takes place at a lower scale generally happens much faster than those at a higher scale. It is now clear that “prostate cancer” admits many descriptions (ways of looking at the system), each of which is only partially true. Each way of looking at a “cancer system” requires its own description, its own mode of analysis, and its own breakdown into different parts.

It is now clear that observing the prostate cancer as a dynamical disease will reveal more about its underlying complex behavioral features. This way of thinking may further help to clarify concepts, interpret new and old experimental data, indicate alternative experiments, and categorize the acquired knowledge on prostate cancer and its precursor lesions. It is encouraging that medicine, biology, psychology, and mathematics continue to contribute together towards a comprehensive understanding of prostate cancer complexity.
